# Students’ reading comprehension level and reading demands in teacher education programs: the elephant in the room?

**DOI:** 10.3389/fpsyg.2024.1324055

**Published:** 2024-02-07

**Authors:** Pelusa Orellana, Mónica Silva, Vicente Iglesias

**Affiliations:** ^1^Facultad de Educación, University de los Andes, Las Condes, Chile; ^2^Escuela de Administración, Pontificia Universidad Católica de Chile, Santiago, Chile; ^3^Escuela de Ingeniería, Pontificia Universidad Católica de Chile, Santiago, Chile

**Keywords:** evaluative study, reading comprehension, higher education, reading assessment, teacher education, Lexile

## Abstract

**Introduction:**

Reading comprehension is considered a key ability for students in teacher education programs.

**Methods:**

Data from 72 students enrolled in a Chilean school of education was used to estimate the contribution of reading proficiency in first-semester academic performance using regression analysis.

**Results:**

Reading comprehension made a significant, albeit modest contribution to predict students’ academic performance, after controlling for their scores in the standardized national admission tests and high-school grades. The students’ average reading level was below the level of text complexity required in their first term and, although by their senior year they had made significant progress in reading comprehension, their reading level continued to be lower than text demands.

**Discussion:**

A qualitative exploration of students’ reading behaviors and attitudes revealed they devoted few hours per week to reading class material and even less time to reading for leisure. Faculty were cognizant of the reading deficits of their students but had few suggestions as to how to address. Future studies in higher education should confirm whether the misfit between reading proficiency and reading demands observed in this school of education is the exception or the rule.

## Introduction

Reading comprehension is crucial for students to succeed in college and become independent learners (De-la-Peña and Luque-Rojas, 2021). Good reading habits are essential for effective communication and expression in academic and professional settings (Cox et al., 2003; Livingston et al., 2015).

Deficits in reading skills are a concern for faculty in many countries (Luyten, 2022). Among other factors, limited high school reading experiences focused on literal comprehension and a lack of exposure to challenging texts may contribute to students’ poor reading habits and skills (Wolfe and Woodwyk, 2010; Livingston et al., 2015; De-la-Peña and Luque-Rojas, 2021). University students recognize the importance of academic reading but admit to not reading enough (Gorzycki et al., 2020). Students enrolled in schools of education are no exception, and research shows they do not come into teacher education programs with high levels of reading competencies or reading habits (Benevides and Peterson, 2010). Applegate et al. (2014) state that almost one half of aspiring teachers will be called upon to inspire their students with a love of reading they do not possess.

Well prepared teachers are essential for successful schooling, given the role they have in helping young children learn to read (Duncan, 2011; Kent et al., 2013). Their preparation to teach reading is crucial, and while conceptual and pedagogical knowledge about teaching reading is necessary (Hikida et al., 2019), they also transmit their own attitudes, beliefs, and experiences as readers (Benevides and Peterson, 2010; Crawford and Reidel, 2022).

Concerns regarding the quality of teacher education programs have prompted investigations into the academic skills of preservice teachers, particularly their verbal ability, which has shown correlations with students’ academic performance (Conaway et al., 2003).

### Reading comprehension assessment

The assessment of reading skills as an entrance prerequisite for higher education is common in most educational systems around the world and is often done via standardized multiple-choice tests. These assessments typically involve reading narrative or informational passages and answering multiple-choice questions that tap into both literal and inferential comprehension abilities. Even though standardized reading comprehension tests have been singled out as having deleterious consequences for student performance and motivation (Pyle et al., 2017; De-la-Peña and Luque-Rojas, 2021), these are widely used at all levels of the educational system. Albeit not perfect measures, when judiciously applied, the information that sound testing practices convey can be beneficial to instruction (Snyder et al., 2005; Sabatini et al., 2014).

The Lexile Framework is a measure of reading comprehension that assesses an individual’s ability to derive meaning from text and determines the complexity level of the text itself (Stenner, 1999). Using the item response theory model, the Lexile Framework provides reading ability scores for students on the same scale as text complexity. This enables the assessment of the alignment between students’ reading ability and the textual complexity they encounter in their first year of college, thus estimating their readiness to handle university-level reading demands (Stenner et al., 2012).

The question arises whether schools of education prioritize and assess students’ reading comprehension as a valuable skill, contributing to its development during their studies. If so, a reading comprehension test should be at least as predictive of academic performance, if not more predictive, than a general standardized admission test tailored to assess curricular learning in secondary education.

In this study, we explored the predictive capacity of a Spanish version of a Lexile-based reading comprehension test developed by MetaMetrics, Inc. (Sanford-Moore et al., 2020) on the academic performance of undergraduates in their first term in a Chilean school of education. We assessed the predictive contribution of this test over and beyond the mandatory standardized national admission tests in Language and Mathematics (PSU-L and PSU-M, similar to the SAT), as well as the students’ high-school grade point average (HSGPA), which were employed for university selection in Chile until 2020.

Evidence regarding the predictive capacity of reading comprehension has relevance, from an institutional perspective, for the improvement of student selection and program advancement. Specifically, we aimed to: (1) assess whether including a reading comprehension measure improved the prediction of early university performance, (2) establish a diagnostic benchmark for reading comprehension at the program’s start, (3) assess changes in student reading comprehension at program completion, and (4) orient instructional changes, if necessary, to monitor and enhance reading comprehension proficiency throughout the program.

## Methods

### Design and participants

The study consisted of a mixed methods research design. As defined by Johnson et al. (2007), it is a type of research that “combines elements of qualitative and quantitative research approaches (e.g., use of qualitative and quantitative viewpoints, data collection, analysis, inference techniques) for the broad purposes of breadth and depth of understanding and corroboration” (p. 123).

Initially the study was conceived as an exploratory quantitative study to guide institutional decision-making to improve the quality of teacher education programs. In view of the findings, we opted to complement the information from the quantitative correlational study (regression analysis) with a qualitative post-hoc probe, via a survey and focus groups to contextualize and gain a deeper understanding of the data.

Data collection took place from March 2019 to September 2022 at a medium-sized private university in Chile, which offered three teacher education programs. In 2022, the school had a total enrollment of 414 students, with over 90% of them admitted through the national centralized admission system. The study sample comprised 72 female students admitted to the programs through the regular 2019 admission process and therefore taking first-year courses. Students were divided among the programs as follows: Early Childhood Education (ECE, *n* = 29), Elementary Education (EE, *n* = 28), and Bilingual Education or English as a Second Language (ESL, *n* = 15). Students were, on average, 19.2 years old when entering the programs:18.9 years old (ECE), 19.2 years old (ESL), and 19.3 years old (EE). In a self-reported questionnaire filled for admission purposes a majority of the sample (70.3%) reported living in one of the five most affluent communes in the nation (Cabrera, 2022) and 80.6% had graduated from a private paid high school. Students’ parental education levels were also high: 82.8% of their mothers and 87.7% of their fathers had attended an institution of higher education.

The qualitative (post-hoc) phase of the study consisted of a survey responded by 62 students, and two focus groups. Nine students and seven instructors participated in the latter. Faculty had a minimum of 3 years of teaching experience and were ascribed to at least one of the three programs and taught classes at the freshmen level.

In the second semester of 2022, students in their final term before graduation were invited to retake the test to assess their reading comprehension. As an incentive to participate, students were offered feedback on their progress. Despite extensive efforts to recruit volunteers, only 15 students agreed to take part in the assessment and 14 successfully completed it.

### Measures

#### Quantitative measures

The criterion variable in the quantitative analysis was the students’ first-term university grade point average (FTGPA) at the school of education.

Only first-term courses were selected as an indicator of achievement because students in the three programs took the same set of courses, while second-term course loads could vary based on courses passed or failed in the first term.

The criterion variable (FTGPA), and the admission predictors–PSU-L and PSU-M test scores, and HSGPA–were obtained from the university’s institutional records. The two Lexile measures were obtained from a Spanish Reading Comprehension assessment developed by MetaMetrics Inc. (Lexile Framework for Reading, n.d.), an online, adaptive comprehension assessment consisting of 40 passages: Students read the passages and answered multiple-choice inferential comprehension questions.

The Spanish Lexile Framework, like the English version, provides a reading measure to estimate a person’s reading ability in Spanish as well as a text measure that estimates the difficulty level of a text in Spanish based on semantic and syntactic complexity. The English Lexile framework was the basis for developing the online Spanish reading assessment used in the current study (Orellana and Melo, 2015). Since the student’s reading ability and text complexity are placed in the same scale, it is possible to estimate the level of fit (or misfit) between them.

**Figure 1:**
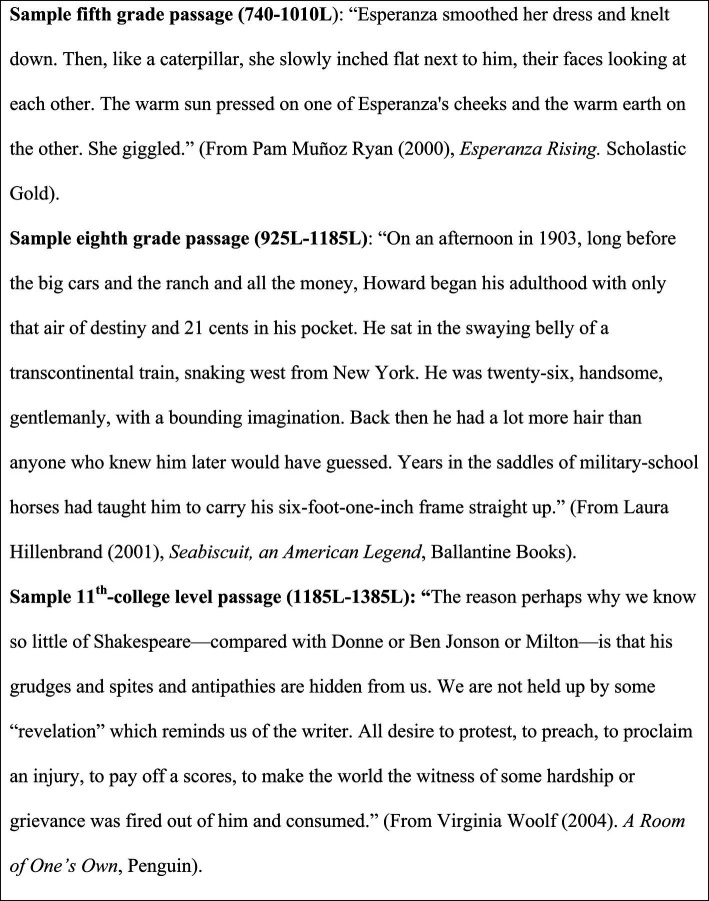
Sample texts and corresponding Lexile measures (Ryan, 2000; Hillenbrand, 2001; Woolf, 2004).

The passages in the text box presented in Figure 1 are examples that vary according to Lexile® levels and were estimated using the software provided by MetaMetrics® to gage the level of text complexity in English (Lexile Text Analyzer, n.d.)^1^.

The first Lexile-based measure employed in the study corresponded to the score obtained by the students in reading comprehension at program entry. Lexile measures range from zero for early readers to above 2000 for advanced readers. In the United States, university-level reading demands typically fall within the range of 1,385 L and above (Williamson et al., 2014).

The second Lexile-based measure–the Lexile Fit Measure–assessed whether the assigned course readings were consistent with the students’ comprehension level. To estimate the Lexile Fit Measure, a sample of assigned readings from the first term was coded and analyzed. The samples included readings from textbooks and articles selected from common course syllabi of the three study programs. One of the authors analyzed the selected readings using a Spanish Lexile analyzer, an online tool similar to English text analyzer that provides an estimate of text complexity based on factors such as sentence length and word frequency (MetaMetrics, n.d.). A positive score in the Lexile fit measure indicated that the student’s reading ability exceeded the demands of the required readings, while negative scores indicated that the reading demands were higher than the student’s level of comprehension. A score close to zero indicated a good fit between the reading demands of the courses and the student’s actual reading ability.

For the regression analysis. The criterion variable (FTGPA) and the admission predictors–PSU-L and PSU-M test scores, and HSGPA–were obtained from the university’s institutional records.

#### Qualitative measures

To better understand the outcomes of the quantitative analyses, we collected additional qualitative data about students’ reading habits for both academic and recreational purposes through a survey instrument followed by a focus group. We also carried out another focus group with faculty members to probe into the findings from the quantitative analyses and student data. Both focus groups were conducted online via Zoom in August 2021, and led by an independent moderator unaffiliated with the university, who also transcribed and analyzed information. Joining the focus groups was voluntary, and separate ethics clearance was secured for the qualitative part of the study.

##### Student survey

Students were requested to complete an online survey adapted from a study by Huang et al. (2016), which was administered to assess students’ reading habits, their perceptions of the importance of reading assignments, and their peers’ reading behaviors. Huang et al. (2016) is a modified version of Chen’s (2007) College Students Reading Habits Survey and contains questions about the amount of weekly time students spent on academic and non-academic activities, as well as their estimates of the percentage of peers who completed reading assignments. Additionally, in our survey we explored students’ perceptions of faculty assessment practices related to assigned course readings. The variables examined via the qualitative analyses included time spent on academic reading, extracurricular reading, part-time jobs, sports/leisure activities, internet use and socializing via Facebook. We added questions to inquire about students’ perceptions regarding their peers’ compliance with assigned academic reading and how academic readings were assessed in class. The online survey was sent to all students in July 2021, and 62 of them answered it (86% response rate).

##### Focus groups

The post-hoc focus groups were conducted in August 2021 via Zoom after the qualitative data was analyzed and the survey was examined. The aim was to deepen the understanding of the findings from both the students’ and faculty perspective. A decision was made to conduct the focus group separately for students and faculty, to avoid social desirability bias and gain insights from their personal standpoints. An independent moderator, unaffiliated with the university, guided the discussions and transcribed the information, under conditions of anonymity.

#### Student focus group

An open invitation was issued to all students to participate in the focus group, expecting to form more than one group. However, only nine students volunteered to participate, four from EE program, four from the ESL program, and one from the ECE program. The primary objective of the focus group was to provide evidence to answer the question of how students were coping with reading demands in the program, given their low reading levels. In addition, we aimed at exploring the participants’ perspectives regarding the role and importance assigned to reading within teacher education programs and to gain insights into their own reading practices and their perceptions of their peers’ reading behaviors. The facilitator also probed into students’ views of the role of instructors in promoting reading.

#### Faculty focus group

After the difficulties experienced in enlisting participants for the student focus group using an open call, we opted for purposive sampling to invite faculty with the ability and capacity to provide relevant insights to understand the phenomenon under study (Morgan, 1988). In that sense, the aim of the focus group was not meant as an instance to build a consensus or generalize conclusions to a broader population of faculty members, but to explore the participants’ perceptions of their students’ reading behaviors and inquire about their efforts and insights as to how to promote effective reading practices.

Seven instructors that met the requirements–i.e. who had at least 3 years of teaching experience at the school of education and who had taught first-year courses–agreed to participate in the faculty focus group and two others declined. Three faculty taught courses in the ECE program and the other four taught both at EE and ESL programs. Like student participants, faculty participated under conditions of anonymity.

##### Quantitative analyses

To estimate the Lexile Fit Measure, a sample of assigned readings from first term courses was coded and analyzed. The samples included readings from textbooks and articles selected from common course syllabi of the three study programs. One of the authors analyzed the selected readings using the Spanish Lexile analyzer, an online tool that provides an estimate of text complexity based on factors such as sentence length and word frequency.

A two-step hierarchical regression was conducted with FTGPA as the criterion variable. The predictors introduced at the first step were three mandatory predictors employed in the national centralized admission system: HSGPA, and scores in the PSU-M and PSU-L tests, respectively. These predictors were entered simultaneously at step one. Three different combinations of reading comprehension measures (Lexile score and Lexile Fit score) were entered in the second step and the change in explained variance from step 1 to step 2 was assessed.

Three regression models were fitted for the full sample since the small sample sizes within programs precluded separate regression analyses. Prior to merging the data sets we tested for differences among the variables in the analysis (see Table 1 in the Results section).

**Table 1:** Means and standard deviations for study predictors and criterion.

	HSGPA^a^	PSU-M^b^	PSU-L^a^	Lexile^a^	Lexile Fit^a^	FTGPA^c^
ECE (*n* = 29)	570 (49)	593 (39)	571 (55)	928 (92)	−271 (93)	5.2 (0.46)
EE (*n* = 15)	603 (55)	611 (29)	581 (62)	954 (117)	−297 (117)	5.5 (0.37)
ESL (*n* = 28)	605 (57)	632 (48)	580 (56)	919 (71)	−332 (71)	5.8 (0.41)
TOTAL	590 (55)	612 (44)	576 (56)	930 (90)	−300 (94)	5.5 (0.52)

Standard Deviations are presented in parentheses. HSGPA, high-school grade point average; PSU-M, score in the national admission test in Mathematics; PSU-L, score in the national admission test in Language; FTGPA, first term grade point average; ECE, Early Childhood Education; EE, Elementary Education; ESL, English as Second Language. ^a^All groups are homogeneous according to Scheffé S procedure (unequal n’s). ^b^Significant difference between ECE and ESL. ^c^Significant difference between ESL and the two other groups (ECE and EE). All significant contrasts reported at *p* ≤ 0.05.

The three models tested included all mandatory predictors (HSGPA, PSU-Language Score and PSU-Mathematics Score) at step 1, but predictors varied at step 2:

Model: Step 1 Step 2

1. Mandatory predictors + Lexile Score
2. Mandatory predictors + Lexile Fit Score
3. Mandatory predictors + Lexile Score and Lexile Fit Score

We also estimated the progress in reading level of students from their first to last term in a subset of the sample. The analysis was conducted on a subset of 15 students who voluntarily retook the test in their final term before graduation. Additionally, we assessed the change in text complexity of required readings between the first term and the last term of studies.

Statistical analyses were conducted using SPSS version 27 (IBM Corp, 2020).

##### Qualitative analyses

We analyzed the survey and focus group in terms of three broad categories of interest: (1) students’ own behaviors relative to reading, (2) students’ perceptions relative to their peers’ behavior, and (3) students’ and their peers’ value of academic reading. For the faculty focus groups the categories of interest were: (1) perceptions of students’ academic reading behavior, (2) value assigned by their students to reading, and (3) instructional activities and suggestions to foster active reading in their courses. We report percentage of responses in the survey but not for the focus groups on two reasons. The first is that through the focus groups we aimed at gaining a deeper understanding of the phenomenon under study via insights that may or not have been widely shared by the participants but helped clarify the nature of the problem and generate alternative ways to address it. The second refers to the small number of students and faculty that took part in the focus groups. With only seven participants, the opinion of a single faculty member would appear overstated when expressed in percentage points. For example, one opinion out of seven represents 14.3% of variation in percentage points, while the same opinion in 50 would represents a variation of two percentage points.

## Results

### Descriptive statistics

The descriptive statistics for the criterion and predictor variables for students enrolled in the three education programs showed few differences at entry between the programs (see Table 1).

No statistically significant differences were observed between students enrolled in the different programs in HSGPA, PSU-L, Lexile scores and Lexile Fit. Differences were observed in PSU-M, with ESL students scoring higher than ECE students (*p*  0.01). ESL students also scored higher than ECE and EE students in FTGPA (*p*  0.01 respectively).

Students exhibited low Lexile scores in the three programs, with average scores ranging from 919 to 954, corresponding approximately to the reading comprehension level of United States fifth graders (Lexile and Quantile Tools, n.d.). The complexity of the assigned first term texts exceeded students’ reading levels, ranging from approximately 1,200 L to 1,250 L (ECE = 1,199 L; EE = 1,251 L; ESL = 1,250 L), appropriate for tenth graders in the United States (Lexile and Quantile Tools, n.d.).

Bivariate correlations between individual predictors and the criterion variable are reported in Table 2.

**Table 2:** Correlations between FTGPA and predictors.*

	ECE (*n* = 29)	EE (*n* = 15)	ESL (*n* = 28)	Total (*n* = 72)
Lexile score	0.31 (0.059)	−0.06 (0.539)	0.01 (0.489)	0.07 (0.274)
Lexile fit	0.30 (0.059)	−0.03 (0.539)	0.01 (0.489)	−0.08 (0.735)
PSU-language	0.31 (0.051)	0.31 (0.132)	0.41 (0.016)	0.31(0.004)
PSU-mathematics	0.05 (0.404)	−0.04 (0.550)	0.06 (0.377)	0.27 (0.011)
HSGPA	0.23 (0.117)	0.74 (0.001)	0.29 (0.070)	0.43 (0.001)

*Exact *p* values in parentheses.

As can be seen from Table 2, correlations ranged from virtually no association (*r* = 0.01) to a high of *r* = 0.74. The magnitude of the correlations, particularly for Lexile Score and Lexile Fit varied across programs, from close to zero to medium-sized correlations according to Gignac and Szodorai (2016), who employ a less stringent criteria than Cohen’s (1988). For the total sample, only correlations between FTGPA and mandatory predictors were significant, and their magnitude corresponded to a medium-large range.

### Regression analyses

We only report the results for the best fitting model (Model 3), for the full sample (*n* = 72), which includes both reading comprehension variables at step 2.

Mandatory predictors at Step 1 explained 25% of the variance. Results at Step 2 showed a significant R-square change, signaling that the addition of Lexile score and Lexile Fit measures improved prediction over and beyond Step 1 predictors. The predictive capacity of the set of mandatory predictors and HSGPA was modest, and the introduction of the reading measures contributed an additional 13%. Total variance explained by the model amounted to 38% (see Table 3), indicating model fit could be improved by the inclusion of additional variables with 62% of the variance still unaccounted for.

**Table 3:** Regression results.

Model	SS	Df	MS	F (p)
Step 1: Mandatory predictors				
Regression	5.83	3	1.79	8.83 (0.000)
Residual	13.82	68	0.20	
Total	19.20	71		
Adj. *R*^2^ Step 1 = 0.25				
Step 2: Mandatory Predictors + Reading Measures				
Regression	8.16	5	1.63	9.75 (0.001)
Residual	11.05	66	0.17	
Total	19.20	71		
Adj. *R*^2^ Step2 = 0.38				

Step 1 predictors: HSGPA, PSU-L and PSU-M. Step 2 predictors: HSGPA, PSU-L, PSU-M, Lexile Score and Lexile Fit Score.

Table 4 reports the values of the coefficients at Steps 1 and 2. At Step 1, two of the three mandatory predictors were significant: HSGPA and PSU-Language. At Step 2 both predictors remained significant, as were Lexile Score and Lexile Fit Score. PSU-Mathematics was not significant neither at the first or second step.

**Table 4:** Coefficients at steps 1 and step 2.

Variable	*B*	SE	Beta (standardized)	*t* (p)
Step 1		
Intercept	0.972	0.949		1.02 (0.310)
PSU-Language	0.002	0.001	0.264	2.52 (0.014)
PSU- Math	0.002	0.001	0.142	1.33 (0.189)
HSGPA	0.004	0.001	0.374	3.54 (0.001)
Step 2				
Intercept	−7.688	2.392		−3.21 (0.002)
PSU-Language	0.003	0.001	0.28	2.746 (0.008)
PSU- Math	7.70 E-5	0.001	0.007	0.63 (0.950)
HSGPA	0.03	0.001	0.28	2.817 (0.006)
Lexile Score	0.08	0.002	1.409	3.752 (0.000)
Lexile Fit Score	−0.08	0.002	−1.519	−4.039 (0.001)

Step 1 predictors: HSGPA, PSU-L and PSU-M. Step 2 predictors: HSGPA, PSU-L, PSU-M, Lexile score and Lexile fit score.

Interestingly, Lexile Score and Lexile Fit were not significant when each was individually added to the regression model (Models 1 and 2, not in table), but when both were simultaneously included in the analysis, they contributed significantly (albeit modestly) to the explanation of the variability in first term achievement. As described by Darlington (1990), suppression effects may be operating since both Lexile Score and Lexile Fit score have a nil zero-order correlation with the criterion and a significant beta coefficient in the regression model when both are included at Step 2. When both variables are in the model, one possibly controls for the sources of error in the other variable, i.e., removing irrelevant variance and thus increasing the contribution to the regression effect. In this case it is likely that Lexile Fit Score may be capturing variability associated to the specific education programs. The result is a stronger and significant relationship between reading comprehension as measured by the two reading variables and FTGPA.

### Reading progress between the first and last term as measured by Lexile scores

Another cogent question was how much students progressed in reading comprehension from the first to the last term. Regrettably only 15 students volunteered to complete the Lexile Scale in 2022 although all 72 participants were repeatedly invited to retake it (approximately 21% response rate). Since self-selection was present, we searched for potential differences between volunteers and non-volunteers in study variables. Using t-test comparisons, no significant differences were observed between both groups as shown in the following table.

In terms of progress in reading comprehension, the students showed a significant improvement in their scores compared to their initial score. Their average reading level increased significantly from 929 L to 1,135 L (paired *t*-test: *t* = 7.41; *p*  0.001). This increase sets the group at approximately the average of U.S eight graders reading comprehension (Lexile and Quantile Tools, n.d.), still below the level of text complexity required in their first term. While there was improvement in students Lexile scores from the first to the last term, text complexity also increased across all three programs during the same period. In the last term, the average text complexity reached 1,490 L, surpassing the average eighth grade reading level of the students. Therefore, the gap between students’ reading comprehension level and the reading demands detected in their first term persisted in their last term of studies.

### Findings *post hoc* study

As mentioned earlier, we expected the Lexile measures to make a larger contribution in explaining the variance of students FTGPA, under the assumption that reading is a basic vehicle for content learning in schools of education (Table 5). With the results from the quantitative study, we had no answers to offer to questions such as: Are students investing efforts in reading? Are faculty promoting reading? What pedagogical practices can facilitate active reading on the part of students? Hence, we opted to collect additional data through a survey followed up by focus group discussions to address these issues.

**Table 5:** Descriptive statistics of study variables for volunteers and non-volunteers.

	FTGPA	Last -term GPA	PSU-L	PSU-M	Initial Lexile score
Non-volunteer (*n* = 57)					
Mean	5,4	5.7	572	609	929
SD	0.56	0.42	51	43	86
Volunteers (*n* = 15)					
Mean	5.6	5.9	595	624	929
SD	0.46	0.36	74	46	100
*t*	0.861	1.64	1.39	1.17	0.07
Value of *p* (2-tailed)	0.392	0.110	0.169	0.245	0.940

### Student survey

The online survey was sent to all students in the sample, and 62 of them answered it (86% response rate). Respondents reported they spent the largest number of hours per week following online classes, studying, and doing homework. Results are presented in Table 6.

**Table 6:** Self-reported weekly time spent on academic and nonacademic activities.

Activities	*M* (SD)
Online classes	8.1 (1.7)
Studying/homework	5.9 (2.2)
Group work	4.8 (2.2)
Academic reading	4.1 (2.3)
Computer	5.3 (1.9)
Sports	2.2 (1.2)
Leisure reading	1.5 (2.0)
Working at paid job	1.5 (2.2)

*n* = 62.

In the survey, students were also asked about their perceptions of their peers’ compliance with assigned academic readings. Approximately 20% of respondents (12/62) estimated that their classmates read all the required materials, while only 3.2% (2/62) believed they read 80% all the assigned material. Twenty-three students (37.1%) also reported it was common practice for students to divide the readings among a group and exchange summaries. Almost 50% of students (30/62) expressed the belief that it was possible to pass their courses with minimal investment in academic reading.

Finally, students were asked about the ways in which instructors assessed academic readings. Students were given several options and could choose more than one. Most respondents (51/62, corresponding to 82.3%) indicated that their instructors employed in-class discussions and quizzes (41/62, corresponding approximately to 66%) to evaluate their comprehension of the readings. However, it is noteworthy that approximately 20% (12/62) of students indicated that their instructors frequently overlooked assessing the assigned readings entirely.

#### Student focus group

We grouped findings taking into account the 3 categories of verbalization we initially defined: (1) students’ own behaviors relative to reading, (2) students’ perceptions relative to their peers’ behavior, and (3) students’ and their peers’ value of academic reading.

Regarding the first category, two topics emerged: first, that there is a change in reading behavior throughout the semester; that is, students express that they make a commitment to read the assigned material and do, in fact, complete required readings at the beginning of the semester, but this commitment wanes as the semester progresses. Some attribute this to the fact that they ascribe more priority to other academic tasks, such as studying, group work, and attending classes. The second topic is the notion that non-academic reading is an activity that one should engage in only during summertime. One student expressed that she read for pleasure 15 to 20 min every night, while four admitted reading occasionally, for example once a week. For the second category, four students acknowledged that they, and many of their peers too, did not read the required readings particularly if assigned texts in English. Division of labor in the form of partial summaries to be shared by a larger group appeared to be a generally accepted form of coping with reading demands. Students who did not incur in the practice did not object the practice but mentioned the lack of trust in the quality of their peers’ summaries as the main reason for abstaining (“often summaries are incomplete or imprecise”).

The topic that emerged for the third category was an overall consensus of the importance that reading has in higher education, yet students also openly acknowledged that they themselves did not commit to it. When asked about the reasons for not completing their academic reading they mentioned lack of time, lack of motivation, difficulty understanding certain texts, and excessive workload in their teacher preparation programs.

#### Faculty focus group

The first category we sought to examine was faculty perceptions of students’ academic reading behavior. Instructors widely agreed that there were two kinds of students: some had a “natural” interest for reading while others had no interest whatsoever, and the latter were the norm. Their estimate was that 10% of their students were interested in reading, 10% who would not read at all, and the remaining 80% would read only if they had no choice. When delving more deeply into the reasons as to why 90% of students did not feel inclined to read, one faculty member expressed that lack of time was one reason invoked as a justification and mentioned that the level of difficulty of required readings in some courses could also affect their lack of interest in carrying out a thorough reading of the text. Some perceived that as the semester progressed, students prioritized other academic activities over reading academic material. For the second category, value assigned by their students to reading, opinions varied very little: if students valued reading only a minority expressed it by spending time in it. Finally, when asked to describe instructional activities and suggestions to foster reading in their courses they described what they did rather than propose novel approaches. Two of the seven participants said they did group work in which students discussed and/or presented the assigned reading. Two other participants said they guided their students’ academic reading by walking them through the structure of a journal article and helping students identify and analyze the information that each section contains. Students would learn to identify research questions and follow the methods section more thoroughly. Four faculty members also mentioned the importance of providing additional context to the readings; for example, if students had to read a book chapter about the Montessori curriculum, faculty members should explain who Maria Montessori was, and why she became involved in education. This strategy, they said, might help students engage more actively in the reading. Finally, regarding the use of quizzes to make sure students completed the readings, 5 out of 7 faculty members said they assessed required readings, but they no longer gave quizzes. Three of them had an “open book” policy; that is, they allowed students to consult the required reading while writing to respond to a prompt that usually required more critical and argumentative writing instead of mere information recall.

## Discussion

This study aimed to determine the relevance of reading comprehension for student success in teacher education programs. The results of this exploratory study showed that reading comprehension measures contributed to predict, albeit modestly, first-term grades beyond standardized test scores and high-school grades. This finding is consistent with international evidence of a small association between performance on reading comprehension assessments and college grades (Clinton-Lisell et al., 2022). The least predictive of the mandatory selection instruments was PSU-M. The absence of Mathematics courses in the first term of teacher education programs may explain the non-significant PSU-M. The absence of Mathematics courses in the first term of this teacher education program may explain the non-significant PSU-M coefficient at both step 1 and step 2. We expected the Lexile measures to make a larger contribution in explaining the variance of students’ FTGPA. The main findings from the student focus group also align closely with international evidence, particularly a study by Gorzycki et al. (2020) that reported that, while students acknowledged the importance of reading, they did not engage in regular reading practices. In other parts of the world, researchers have expressed concerns about deficient reading habits among university students (Gorzycki et al., 2016, 2020; Alsaeedi et al., 2021). However, the magnitude of the gap between students’ average reading levels (akin to fifth graders) and the reading demands in their first term was unexpected and should be a matter of concern not only for the institution but also for public policy. Chile has been recognized as a country where socio-economic background strongly influences student performance (Guthrie et al., 2019). Given that the teacher programs under study attract students from private high-schools–that have more resources and provide a more favorable educational background that translates into higher student performance in mathematics and verbal tests compared to public schools (Agencia de la Calidad de la Educación, 2023) it is likely that deficits in undergraduates’ reading comprehension may be widespread.

While it was encouraging to observe that students’ reading comprehension skills improved over the four-year teacher preparation program, faculty and university officials wondered about strategies to stimulate progress. Students who volunteered to retake the reading comprehension assessment in their last term before graduation improved their reading ability by roughly 206 L, but this mark is still well below the demands of undergraduate texts. Even if one assumes that the increase in reading comprehension proficiency generalizes across students, the growth appears insufficient to deal with increasingly complex texts, ranging from 1,200 L to 1,500 L.

On the other hand, as online reading becomes a trend the evidence indicates that digital reading does not contribute to improve reading comprehension in students as traditional print reading does (Altamura et al., 2023). A previous meta-analysis by Clinton (2019) also identified text modality as a factor that can negatively affect reading comprehension. Trakhman et al. (2019) concluded that students read more quickly when engaged digitally, but their actual performance in terms of comprehension was substantially better when reading in print. Thus, the increasing use of digital reading may not favor a spontaneous improvement of reading comprehension in undergraduates.

The post-hoc probe revealed that students report they devote scant time to academic and leisure reading and complain that required readings are difficult and unmotivating. There is a consistent perception among both students and faculty that only a minority of students read all the assigned course material. The easy path for faculty and administrators is to diffuse responsibility and rationalize that someone else has to address the problem (e.g., “one cannot do much because students come into the program with a negative and deeply ingrained attitude forged previously during their high-school years”). However, such a deterministic outlook curbs the possibility of change. If reading comprehension ability improves with reading frequency and reading volume in print, faculty should be focused on how to help these future teachers invest time and effort to develop it. An encouraging finding was that faculty acknowledged the problem and recognized how students circumvent reading requirements, although they offered limited suggestions on how to address it.

### Study limitations

In addition to the inherent limitations of an exploratory correlational study such as this one, and the fact that it was not possible to test separate regression models for the three programs due to the low ratio of observations to predictors within them, two other limitations surfaced as the study progressed. The first was that not all students agreed to take the Lexile Scale assessment in their final term, despite the efforts displayed by the faculty toward that end. Although there were no significant differences observed between volunteers and non-volunteers in study variables it is not possible to rule out that respondents may differ from non-respondents in other uncontrolled variables that may eventually influence the outcome of interest.

A second challenge to the generalizability of the findings is the historical context in which it was conducted. It is not possible to determine the extent to which the pandemic influenced the findings. The Covid-19 pandemic forced higher education institutions and teacher education programs into a remote teaching mode which required new ways of addressing teaching and learning needs (Carrillo and Flores, 2022). The students’ self-reported reading behaviors may have been influenced, either positively or negatively, by the pandemic context. While the measures taken in 2019, such as high-school grades, admission test scores, and initial Lexile measurement, were not affected by quarantine restrictions, the university experience between 2020 and 2022 was likely impacted by it. Thus, the observed increase in reading comprehension, although seemingly minor, was achieved under unfavorable circumstances due to the COVID-19 pandemic, and it may underestimate the actual progress students would make under normal conditions. Consequently, caution is advised when interpreting the reported results for institutional decision-making and generalizing these findings to the broader population in a post-pandemic scenario.

### Moving forward

To move forward it is necessary to implement a culture of evaluation and outcomes assessment in reading skills inside schools of education and undergraduate programs. Future studies should confirm whether the misfit between reading proficiency and reading demands observed in this school of education is the exception or the rule.

Institutional research is needed but also the willingness of university officials to share information about the challenges and successful remedial interventions. Quasi-experimental research is needed to inform about successful (and unsuccessful) interventions to promote reading comprehension.

The assessment of reading level at program entry is a necessary first step to develop a strategic plan to progressively improve reading practice. As in other parts of the world, our data shows a vast proportion of students perceive they can pass courses without reading and while faculty are aware of the problem, they do not engage in academic reading pedagogies (Desa et al., 2020). Decreases in reading abilities can be associated, to some extent, to uncontrollable factors, such as increases in online chatting (Luyten, 2022) and an excess of social networking (Alsaeedi et al., 2021), among others. Still, the fact that it may be a complex, multi-causal and widespread problem does not exempt schools of education from the responsibility in addressing it.

The improvement of reading practices will require a collaborative effort from all parties involved: faculty, students, and university officials. Students lack of concern for their reading proficiency–as evidenced by their lack of interest in retaking the Lexile test in their final term despite extensive efforts by the faculty to incentivize their participation in the assessment–will need to be addressed.

In addition to students’ indifference, another major obstacle to overcome is the tendency to ignore, downplay or simply disown the problem either by portraying it as a cultural and worldwide phenomenon or by shifting responsibility onto other institutions. Acknowledging and confronting the “elephant in the room” is necessary to advance academic reading. Pretending the elephant does not exist is the worst option.

From an institutional perspective, early diagnosis of reading skills (i.e., at program entry) can serve to target students who may need support to cope with first term academic reading demands and also to guide a revision of course syllabi to tailor the reading complexity of texts to match students’ reading level. Syllabi revision can facilitate progress by reducing the initial gaps between reading demands and student’s reading level and also contribute to improve student motivation to read (Frankel et al., 2016).

Finally, simplistic solutions based on a punitive approach to reading, e.g., imposing a mandatory and intensive “read and quiz” policy may result in increased compliance with academic reading but can also further detract from students’ inclination to read. By taking ownership of the “elephant” in a positive way, schools of education can play a vital role in promoting good reading habits among future teachers who can in turn serve as role models for their own students and inspire in them a genuine appreciation of reading.

## Data availability statement

The raw data supporting the conclusions of this article will be made available by the authors, without undue reservation.

## Ethics statement

The studies involving humans were approved by Comité Etico Científico Universidad de los Andes. The studies were conducted in accordance with the local legislation and institutional requirements. The participants provided their written informed consent to participate in this study.

## Author contributions

PO: Investigation, Methodology, Writing – original draft, Writing – review & editing. MS: Formal analysis, Methodology, Writing – original draft, Writing – review & editing. VI: Formal analysis, Investigation, Methodology, Writing – review & editing.

## Footnotes

^1^METAMETRICS^®^, and LEXILE^®^ are registered trademarks of MetaMetrics, Inc. in the United States and abroad. Copyright ©2024 MetaMetrics, Inc. All rights reserved.
